# Exploration of genes associated with induction of the viable but non-culturable state of *Campylobacter jejuni*

**DOI:** 10.1007/s00203-024-03980-y

**Published:** 2024-05-15

**Authors:** Yurina Ohno, Md. Matiur Rahman, Hiroe Maruyama, Yasuo Inoshima, Ayaka Okada

**Affiliations:** 1 Laboratory of Food and Environmental Hygiene, Joint Department of Veterinary Medicine, Faculty of Applied Biological Sciences, Gifu University, 1-1 Yanagido, Gifu, 501-1193 Japan; 2 Department of Medicine, Faculty of Veterinary, Animal and Biomedical Sciences, Sylhet Agricultural University, Sylhet, Bangladesh; 3 Nagaragawa Research Center, API Co., Ltd, Gifu, Japan; 4 Education and Research Center for Food Animal Health, Gifu University (GeFAH), Gifu, Japan; 5 Joint Graduate School of Veterinary Sciences, Gifu University, Gifu, Japan

**Keywords:** *Campylobacter jejuni*, Internal control gene, Microarray, Viable but non-culturable state

## Abstract

*Campylobacter jejuni* is known to enter a viable but non-culturable (VBNC) state when exposed to environmental stresses. Microarray and quantitative real-time polymerase chain reaction (qPCR) analyses were performed to elucidate the genes related to the induction of the VBNC state. The *C. jejuni* NCTC11168 strain was cultured under low-temperature or high-osmotic stress conditions to induce the VBNC state. mRNA expression in the VBNC state was investigated using microarray analysis, and the gene encoding peptidoglycan-associated lipoprotein, *Pal,* was selected as the internal control gene using qPCR analysis and software. The three genes showing particularly large increases in mRNA expression, *cj1500*, *cj1254*, and *cj1040*, were involved in respiration, DNA repair, and transporters, respectively. However, formate dehydrogenase encoded by *cj1500* showed decreased activity in the VBNC state. Taken together, *C. jejuni* actively changed its mRNA expression during induction of the VBNC state, and protein activities did not always match the mRNA expression levels.

**Supplementary Information:**

The online version contains supplementary material available at 10.1007/s00203-024-03980-y.

## Introduction

*Campylobacter jejuni* is one of the leading foodborne pathogens worldwide (Kaakoush et al. 2015), and *C. jejuni* and *C. coli* are the most common bacteria implicated in incidents of food poisoning reported since 2003 in Japan according to the Food Poisoning Statistics of the Ministry of Health, Labour, and Welfare in Japan, (Vetchapitak and Misawa 2019). *Campylobacter jejuni* infection causes symptoms such as abdominal pain, diarrhoea, and fever (Skirrow 1977). The severity of *Campylobacter* infections makes it a public health concern, with potential complications such as Guillain–Barré syndrome, a rare but serious neurological disorder, being linked to the bacterium (Johnson et al. 2017). Additionally, the increasing antibiotic resistance observed in *Campylobacter* strains underscores the need for ongoing research to understand and mitigate this resistance (Man 2011). The primary routes of contamination involve the consumption of contaminated food, particularly undercooked poultry, unpasteurised milk, and unsterilised water (Neimann et al. 2003). Cross-contamination during food handling and inadequate hygiene practices contribute to the spread of *Campylobacter* infections (Neimann et al. 2003).

*C. jejuni* is a spiral-shaped, gram-negative, microaerophilic bacterium (Humphrey et al. 2007). When *C. jejuni* is subjected to environmental stresses such as low temperature, high osmotic stress, nutrient starvation, or unsuitable aerobic conditions, it enters a viable but non-culturable (VBNC) state (Yagi et al. 2022). In the VBNC state, *C. jejuni* is transformed from the normal spiral form into a coccoid form under unfavourable environmental conditions (Santos et al. 2023). Despite the lack of clear evidence to prove that the VBNC state of *C. jejuni* can directly cause human diseases, the risks to food safety and public health cannot be ignored (Chaisowwong et al. 2012). Transformation to the VBNC state under unfavourable environmental conditions has been reported in other bacteria such as *Vibrio harveyi* (Li et al. 2017), *Salmonella* Enteritidis (Morishige et al. 2013), and *Legionella pneumophila* (Ducret et al. 2014), and these bacteria can be resuscitated when the environmental stress is removed. Resuscitation is a complicated process, and the conditions required for it differ among bacteria. Previous studies showed that *C. jejuni* could be resuscitated by incubation with embryonated chicken eggs (Cappelier et al. 1999), in vivo passage in mice (Baffone et al. 2006), incubation with the human colon adenocarcinoma cell line Caco-2 cells (Chaisowwong et al. 2012), and co-culture with amoebas (Axelsson-Olsson et al. 2005). However, the factors that directly trigger resuscitation remain unclear. *C. jejuni* in the VBNC state cannot be detected using culture tests, which are usually used in epidemiological surveys, and undetectable *C. jejuni* in the VBNC state may cause foodborne illnesses. Therefore, understanding the factors that promote resuscitation and developing a culture medium that can detect *C. jejuni* in the VBNC state is essential.

However, the molecular mechanisms underlying the transition to the VBNC state in *C. jejuni* have not been studied in detail. During the VBNC state, the gene expression of *C. jejuni* has been reported to change; for instance, *CadF,* which mediates the binding of *C. jejuni* to fibronectin*,* was shown to be expressed at high levels in the VBNC state (Patrone et al. 2013), indicating the need to clarify the changes in gene expression during the transition from the culturable state to the VBNC state. Therefore, we aimed to identify the genes involved in the transition from the culturable state to the VBNC state in *C. jejuni* by analysing their mRNA expression.

In this study, microarray analysis was performed using RNA derived from *C. jejuni* in both culturable and VBNC states to explore the genes associated with the induction of the VBNC state. Subsequently, quantitative real-time polymerase chain reaction (qPCR) was performed to confirm the microarray results. qPCR is considered the standard method for sensitive measurement of gene expression. First, an internal control gene was selected based on the microarray data to develop an accurate qPCR method. Next, qPCR was performed to verify the changes in the expression levels of differentially expressed genes (DEGs) selected from the microarray results. To reduce the number of DEGs in the microarray, two VBNC conditions were used for induction: low-temperature and high-osmotic stress. Finally, we investigated whether the changes in mRNA expression affected protein activity.

## Materials and methods

### Bacterial strain

*C. jejuni* NCTC11168 strain was purchased from the American Type Culture Collection (ATCC, 700,819, Manassas, VA, U.S.A.). *C. jejuni* was stored in Brucella broth (BD Biosciences, Billerica, MA, U.S.A.) containing glycerol (Fujifilm Wako Pure Chemical, Osaka, Japan) at a concentration of 16% (v/v) at − 80 °C until use.

*C. jejuni* from the stock maintained at − 80 °C was cultured on modified charcoal-cefoperazone-deoxycholate agar (mCCDA) (Oxoid, Basingstoke, Hampshire, U.K.) with CCDA selective supplement (Oxoid). The culture was incubated at 37 °C for 48 ± 2 h under microaerobic conditions generated using an AnaeroPack-MicroAero (Mitsubishi Gas Chemical, Tokyo, Japan) in an anaerobic jar (Mitsubishi Gas Chemical). A single colony was suspended in 15 mL of Brucella broth and cultured at 37 °C for 30 ± 1 h under microaerobic conditions with shaking at 130 rpm using an Innova 4300 Incubator Shaker (New Brunswick Scientific, Enfield, CT, U.S.A). Liquid cultures were then resuspended in fresh Muller–Hinton (MH) broth (BD Biosciences) at a 1:100 dilution (v/v) and cultured at 37 °C for 24 h under microaerobic conditions with shaking at 130 rpm.

The liquid cultures were centrifuged at 8000×*g* for 10 min at 4 °C (these conditions were used in all centrifugation steps described below) in an R12A2 rotor (Hitachi Koki, Tokyo, Japan) using a himac CR20GII centrifuge (Hitachi Koki), and the bacterial pellet was washed with phosphate-buffered saline (PBS) at pH 7.4 and centrifuged again. The pellet was diluted with MH broth and adjusted to an optical density of 1 at 600 nm (OD_600_), corresponding to approximately 1×10^8^ colony-forming units (CFU)/mL. This suspension was used as the control.

### Induction of the VBNC state in C. jejuni

To induce the VBNC state, 10–15 mL of bacterial solution was subjected to two stress conditions: low-temperature (Yagi et al. 2022) and high-osmotic stress (Lv et al. 2019), in accordance with previous reports. Under low-temperature conditions, bacterial cells prepared using the same method as the control samples were incubated at 4 °C under aerobic conditions for 25 days with shaking at 130 rpm. Under high-osmotic conditions, bacterial cells were prepared by suspending cells in 7% NaCl/distilled water (DW) instead of MH broth and incubated at 37 °C under microaerobic conditions.

Culturable cell counts were performed by plating 0.1 mL of the serially diluted suspensions in PBS in duplicate on mCCDA plates. Viable cells were enumerated using a LIVE/DEAD BacLight Bacterial Viability Kit (Thermo Fisher Scientific, Waltham, MA, U.S.A.) according to the manufacturer’s instructions. Ten microliters of the sample solution in 7% NaCl/DW or MH broth were mixed with 10 µL of mixture (1:1) of SYTO9 and propidium iodide in the kit, and the cells were stained in the dark for 15 min at room temperature. The cells in 7% NaCl/DW were confirmed to have the same viability as the cells resuspended in PBS. After incubation, the stained cells were mounted on glass slides and examined using an epifluorescence microscope (BZ-X810, KEYENCE, Osaka, Japan). Viable cells were estimated by counting cells with green fluorescence and without red fluorescence, and non-viable cells were estimated by counting cells with red fluorescence. Cell counting was conducted in four random fields at 400×magnification using two replicates for each sample. Bacterial and viable cell counts were performed every five days under low-temperature conditions and at 0, 5, and 24 h under high-osmotic conditions.

### RNA extraction

The bacterial suspension (1.5 mL and 4 mL of suspensions were used for the low-temperature condition and high-osmotic stress condition, respectively) was mixed with two-fold volumes of RNAprotect Bacteria Reagent (Qiagen, Venlo, Netherlands). The mixture was vortexed, incubated at room temperature for 5 min, and centrifuged at 8000×*g* for 10 min at 4 °C. The precipitate was air-dried for several minutes and stored at – 80 °C until use. Bacterial RNA was isolated from each precipitate using 600 µL of TRIzol Reagent (Thermo Fisher Scientific) and Direct-Zol RNA Miniprep (Zymo Research, Orange, CA, U.S.A.) in accordance with the manufacturer’s instructions. Genomic DNA contamination was eliminated by treating the samples with DNase I (Zymo Research) from a Direct-Zol RNA Miniprep kit. The eluted RNA was quantified using a NanoDrop Lite spectrophotometer (Thermo Fisher Scientific) and stored at − 80 °C until use. For quality control, RNA purity was evaluated using OD 260/280 ratio, and values of 1.7–2.0 were considered indicative of relatively pure RNA. RNA integrity number (RIN) was analysed using an Agilent RNA 6000 nano kit (Agilent, Inc., Santa Clara, CA, U.S.A.) on an Agilent 2100 Bioanalyzer (Agilent), and RNA with RIN greater or equal to 8 was used for microarray.

### Microarray analysis

To explore mRNA expression in the VBNC state, RNA was obtained from nine samples, including three control samples, three low-temperature samples cultured for 15 days, and three high-osmotic stress samples cultured for 24 h. Although 25 days were needed to induce all cells into the VBNC state under low-temperature, 90% of cells had already been induced into the VBNC state on day 15 and we suspected that long time incubation after induction of VBNC state might affect the RNA quality; thus, we used the cells cultured for 15 days. Customised microarray slides (8×15 K) were designed using eArray (https://earray.chem.agilent.com/earray, accessed on 2 February2024) with 8–9 probes/target based on the genome data of *C. jejuni* NCTC 11168 strain. RNA from the control, low-temperature, and high-osmotic stress samples was analysed using the SurePrint G3 Custom-Gene Expression Platform (Agilent). Microarray analysis and data normalisation were performed by Macrogen (Tokyo, Japan) according to the method described in the Agilent manual.

Raw data were extracted using Agilent Feature Extraction Software (v11.0.1.1). Raw data for the different probes of the same gene were then summarised automatically using the Agilent feature extraction protocol to generate a raw data text file, which provided the expression data for each gene probed on the array. Array probes with Flag-A were filtered out. The selected gProcessedSignal values were logarithmically transformed and normalised using the quantile method. The statistical significance of the expression data was determined using the fold change and independent* t*-test, applying the null hypothesis that there was no difference among the groups. The false discovery rate was controlled by adjusting the* p*-value using the Benjamini–Hochberg (BH) algorithm. For the DEG set, hierarchical cluster analysis was performed using complete linkage and Euclidean distance as similarity measures. All data analyses and visualisations of DEG were conducted using R 3.3.2 (https://www.r-project.org, accessed on 2 February2024).

### Primer design and confirmation of the reaction efficiencies

For each gene, primers were designed using the Primer BLAST software (National Center for Biotechnology Information, Bethesda, MD, U.S.A.) (http://www.ncbi.nlm.nih.gov/tools/primer-blast, accessed on 2 February 2024). The primer sequences are listed in Table 1. All primers were confirmed by qPCR to have reaction efficiencies greater than 90%. Reaction efficiencies were determined using the following procedure: The cDNA suspension of the control sample was serially diluted tenfold. For each primer, the cycle threshold (Ct) values and the corresponding DNA concentrations were plotted on a logarithmic scale. The reaction efficiencies were calculated from the linear regression curve of the data points using the following equation:

**Table 1:** Primers used for quantitative real-time polymerase chain reactions and functions of the target genes

Genes	Forward (5′–3′)	Reverse (5′–3′)	Efficiency (%)	Function
Internal control				
* Pal*	TACTTCAATCGCAGCTCTTGC	TCCACCTGAACCACGATTTG	98.6	Peptidoglycan-associated lipoprotein
Target genes				
* cj1500*	GCTGTAGCTGTGGGAGCATT	AAGTCCAACCACACTCGCAA	96.5	Formate dehydrogenase
* cj1254*	CCTTTCTCAAACCAAAATTCAAGCC	ATGGCTTCTAAGCGAGGGTG	95.8	G: T/U mismatch glycosylase
* cj1040*	GGTTTTCCATGGGGTGGAGT	CCATTGTCCTTGTGCTGCAAT	95.2	MFS transport protein
* cj1031*	ACCTTGCCCTAAATGCAATCAC	TTAGGGCTAAGTCCTGCGTC	91.8	cmeD/drug metabolic transporter
* cj0077*	AGGATCTAGGGTGCAAGGGG	AACAACAACTTCAGCTGTGCAA	99.6	cdtC/a cytolethal distending toxin

*E* = – 1 + 10 ^( – 1 /slope)^.

### cDNA synthesis and qPCR

cDNA was synthesised using the PrimeScript RT Master Mix (Takara Bio, Kusatsu, Japan) according to the manufacturer’s instructions. qPCR was performed in 96-well plates at the following condition: each well contained 300 nM each of forward and reverse primers, PowerUp SYBR Green Master Mix (Applied Biosystems, Foster City, CA, U.S.A.), and 11.25 ng of cDNA. qPCR was performed using a StepOne Plus analytical thermal cycler (Applied Biosystems) according to the protocols suggested by Applied Biosystems. The program was as follows: 50 °C for 2 min and 95 °C for 2 min for initial heat activation, followed by 40 cycles of 95 °C for 3 s for denaturation and 60 °C for 30 s for annealing and extension. After amplification, melting curve analysis was performed to verify the specificity of the reaction. Relative mRNA expression levels were compared to the control and evaluated using the ΔΔCt method (Yuan et al. 2006).

### Selection of the internal control gene

The selection of an internal control gene for qPCR was conducted in accordance with previous reports (Rahman et al. 2021, 2023) with some modifications. A total of six genes from the raw microarray data were selected for the candidate internal control gene assessment. The selection was carried out on the following five criteria: (1) fold change ratio between –1.2 and 1.1; (2) expression level exceeding 8000 in all samples; (3) coefficient of variation less than 0.25; (4) detectable in all samples; (5) no significant difference among all samples (*p* > 0.05).

To verify the stability of the genes, qPCR was conducted using 12 samples; six control, two low-temperature, and four high-osmotic stress samples (Table S1). The stability of the putative candidate internal control genes was analysed using five algorithmic software programs; geNorm (Vandesompele et al. 2002), NormFinder (Andersen et al. 2004), BestKeeper (Pfaffl et al. 2004), ΔCt algorithm (Silver et al. 2006), and RefFinder (Xie et al. 2012). In brief, the geNorm algorithmic software was employed to determine the potential stability values (M) of internal control genes, with stable genes being rated based on these M values (Vandesompele et al. 2002). A smaller value of M indicates greater gene stability, while a larger value of M indicates lesser gene stability. A putative internal control gene that is stable should possess an average geNorm M value that is greater than or equal to 1.0. The NormFinder algorithmic software was employed to identify potential internal control genes using a model-based approach utilizing the 2^−delta Ct^ equation, where delta Ct = Ct sample-Ct min (Ct sample is the raw Ct value and Ct min is the least raw Ct value) equation to convert raw Ct measurements into relative quantities (Andersen et al. 2004). On the other hand, BestKeeper algorithmic software was estimated the gene expression variation for each unique putative internal control gene using their Ct values. The assessment involved evaluating the coefficient of variance (CV), standard deviation (SD), calculation of probability (p), and Pearson correlation coefficient (r) for each combination (Pfaffl et al. 2004). This algorithmic software determined the most stable putative internal control gene involves the calculation of the gene's r-value. In the ΔCt algorithmic software, the stability of the putative internal control gene was assessed using the mean SD in the Ct (Silver et al. 2006). When the SD of a gene is low, it is considered to be stable and when it is high, it is considered less stable, suggesting that the gene could serve as a potential internal gene. Finally, the RefFinder algorithm was applied to rank all possible internal control genes by combining the results of four separate algorithmic software (Xie et al. 2012). After the assessment, candidate internal control genes were narrowed down from six genes to three genes. To further refine the selection and identify the most suitable internal control gene, an additional set of ten samples was prepared under the control condition, followed by qPCR analysis using the method described above (Table S2). The most stable gene was used as the internal control for qPCR.

### Enzyme assays

Formate dehydrogenase (FDH) and fumarate reductase (FRD) assays were conducted using a benzyl viologen-coupled colorimetric assay in accordance with the procedures outlined in previous reports (Guccione et al. 2010; Shaw et al. 2012). All assays were performed using the GeneQuant 100 spectrophotometer (GE Healthcare, Chicago, IL, U.S.A.). Protein concentration was determined using the Lowry assay with the RC DC Protein Assay Kit II (Bio-Rad, Hercules, CA, U.S.A.).

FDH assays were performed using bacterial solutions from control samples and samples in the VBNC state induced under low-temperature conditions for 25 days. The bacterial solution (5 mL) was spun down at 8000×*g* for 10 min at 4 °C and then washed twice with 1 mL of 25 mM phosphate buffer (PB). The bacterial cells were subsequently resuspended in 1 mL of 25 mM PB. The final assay mixture consisted of 25 mM PB, 1 mM benzyl viologen, and 10 mM sodium formate. Liquid paraffin was added as a protective layer to prevent reactions with atmospheric oxygen. The assay was initiated by injecting sodium formate solution into the mixture. The reaction was monitored at 578 nm until the absorbance exceeded 2.0. The FDH activity was calculated by determining the nmol of viologen reduced per min per mg of protein.

The FRD assays were performed using bacterial solutions similar to those used in the FDH assays. The final assay mixture contained PBS, 1 mM benzyl viologen, and 5 mM sodium fumarate. A layer of liquid paraffin was added to prevent oxygen from entering the mixture. After adding cells to the buffer and viologen mixture, aliquots of freshly prepared sodium dithionite solution as a reducing agent were injected into the cuvette until a steady absorbance at 578 nm was achieved. The assay was initiated by injecting sodium fumarate solution. The reaction was monitored at 578 nm until the absorbance reached zero.

### Statistical analysis

**Fig. 1:**
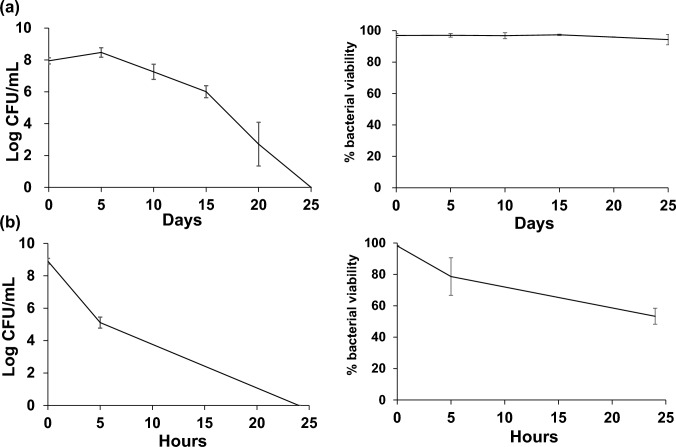
Culturability (left panels) and viability (right panels) of the VBNC state of *C. jejuni* induced under low-temperature (**a**) and high-osmotic stress (**b**) conditions. *C. jejuni* was cultured at 4 °C in MH broth under aerobic conditions for 25 days or in 7% NaCl/distilled water at 37 °C under microaerobic condition for 24 h. Culturable cell counts were performed by plating 0.1 mL of the serially diluted suspensions on mCCDA plates. Viable cells were enumerated using a LIVE/DEAD BacLight Bacterial Viability Kit and indicated as the percentage of live cells per total cells. Bacterial and viable cell counts were performed every 5 days under low-temperature conditions and at 0, 5, and 24 h under high-osmotic conditions. The data show the mean ± SE of three independent experiments. CFU, colony-forming units; mCCDA, modified charcoal-cefoperazone-deoxycholate agar; MH, Muller–Hinton; SE, standard error; VBNC, viable but non-culturable

All experiments were conducted with at least three independent replicates, and data were presented with standard error (SE). The data were analysed for significance using Student’s *t*-test for Figs. 1, 5, or a linear mixed-effects model for Fig. 4. All statistical analyses were performed using EZR (Kanda 2013). The corrected* p*-value cut-off was set at 0.05.

## Results

### Bacterial viability and culturability

The VBNC state was induced under low-temperature and high-osmotic stress conditions, as shown in Fig. 1. Under low-temperature conditions, cells were incubated at 4 °C under aerobic conditions in MH broth for 25 days, and culturable cell counts gradually decreased, reaching zero on day 25 (Fig. 1a, left panel), whereas bacterial viability was 95% until day 25 (Fig. 1a, right panel). Under high-osmotic stress conditions, cells were incubated at 37 °C under microaerobic conditions in 7% NaCl/DW for 24 h, and culturable cell counts rapidly decreased to 10^5^ CFU/mL within 5 h and reached zero at 24 h. The bacterial viability at 5 h and 24 h decreased to approximately 75% and 55%, respectively (Fig. 1b), indicating that approximately 25% (5 h) and 45% (24 h) of the cells were dead; however, the remaining live cells entered the VBNC state in high-osmotic stress condition.

### Selection of the internal control gene for qPCR

An internal control gene was selected based on the results of microarray analysis. To select the internal control gene with the most stable expression, five criteria were applied, as described in the Materials and Methods section.

**Fig. 2:**
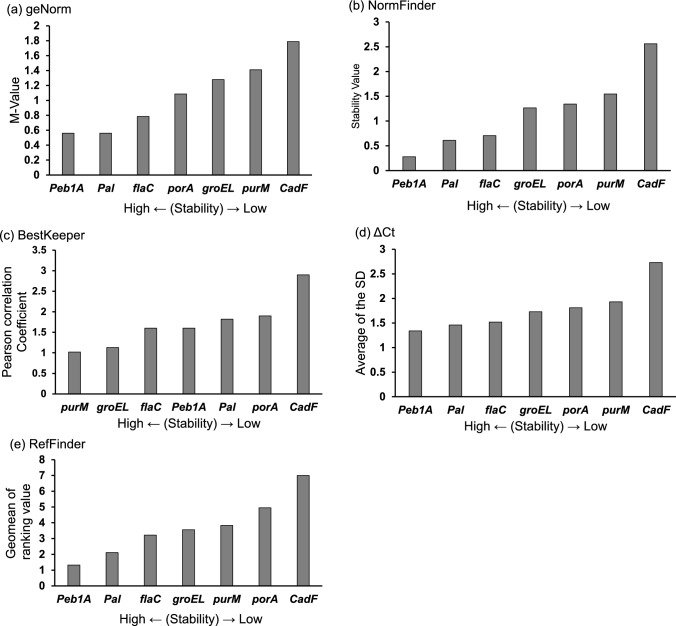
Selection of the internal control gene from six genes. The stability of expression of the six genes selected based on five criteria was measured. qPCR was performed using seven controls, two low-temperature, and four high-osmotic stress samples. The results were analysed by geNorm (**a**), NormFinder (**b**), BestKeeper (**c**), ΔCt (**d**), and RefFinder (**e**). The smaller each value is, the higher the stability. *Peb1A*, *Pal,* and *flaC* were selected as the three most stable genes. qPCR, quantitative real-time polymerase chain reaction

The results were analysed using five different software programs: geNorm, NormFinder, BestKeeper, ΔCt algorithm, and RefFinder. The geNorm algorithm was used to measure the gene expression stability (M-value). Genes with the lowest M-values showed the most stable expression. The results of the geNorm analysis revealed that the stability ranking of the candidate internal control genes were *Peb1A*, *Pal*, *flaC*, *porA*, *groEL*, *purM*, and *CadF*. From the result, it was indicated that the M-value of *Peb1A* was the lowest, followed by *Pal* and *flaC,* suggesting that *Peb1A*, *Pal*, and *flaC* had the most stable expression (Fig. 2a).

The NormFinder algorithm was used to assess the stability of candidate internal control genes. Lower stability values indicate higher stability. According to the NormFinder analysis the stability ranking of the candidate internal control genes were *Peb1A*, *Pal*, *flaC*, *groEL*, *porA*, *purM*, and *CadF*. From the result, the most stable internal control gene identified by this program was *Peb1A*, followed by *Pal* and *flaC*, consistent with the results obtained using geNorm (Fig. 2b).

The BestKeeper algorithm was used to calculate SD and Pearson’s correlation coefficient. A low Pearson’s correlation coefficient indicated a highly stable candidate internal control gene. The BestKeeper algorithm demonstrated the stability ranking of the candidate internal control genes were *purM*, *groEL*, *flaC*, *Pal*, *Peb1A*, *porA*, and *CadF*. The result indicated that the highly stable internal control genes were *purM*, *groEL*, *flaC*, and most less stable internal control genes were *CadF* (Fig. 2c).

The ΔCt algorithm was used with the average of SD values to assess the stability of the candidate mRNAs. A lower SD value indicated a higher number of stable genes. The results revealed that the stability ranking of the candidate internal control genes were *Peb1A*, *Pal*, *flaC*, *groEL*, *porA*, *purM*, and *CadF*. The results also indicated that the candidate internal control gene with the most stable expression was *Peb1A*, followed by *Pal* and *flaC* (Fig. 2d).

**Fig. 3:**
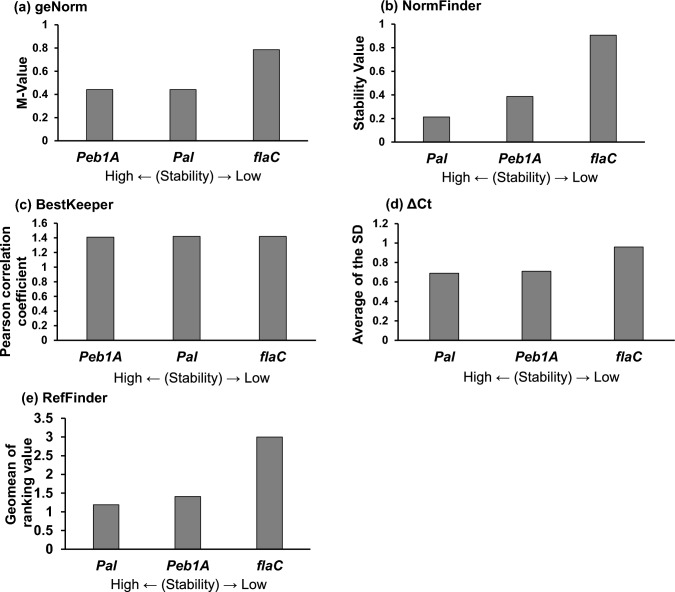
Selection of the most suitable internal control gene from three genes. The stability of the expression of three genes was measured. qPCR was performed using 10 control samples. The results were analysed by geNorm (**a**), NormFinder (**b**), BestKeeper (**c**), ΔCt (**d**), and geomean of ranking value by RefFinder (**e**). The smaller each value is, the higher the stability. qPCR, quantitative real-time polymerase chain reaction

The different algorithms, including geNorm, NormFinder, BestKeeper, and ΔCt were revealed in the different candidate internal control genes. Therefore, the RefFinder program was used to determine the overall ranking of the potential internal control gene candidates. The three most stably expressed genes were *Peb1A*, *Pal*, and *flaC* (Fig. 2e). To further refine the selection, 10 control samples were newly prepared, and additional qPCR was performed (Fig. 3). The results of the analysis of the four software programs were ranked by RefFinder, and *Pal* was identified the most stable gene and was used as an internal control.

### Selection of common DEGs between the VBNC states induced by low-temperature and high-osmotic stress conditions as target genes

The genes whose fold changes ranged between − 1.5 and 1.5 and raw *p*-values were less than 0.05 in microarray analysis are listed in Table S3 (low-temperature condition) and Table S4 (high-osmotic stress condition). Genes predicted to be involved in VBNC induction were defined as target genes and selected as follows. (1) The samples with significant differences between the control and VBNC samples (*p* < 0.05) were selected. First, *p*-values adjusted using the BH algorithm were used to select target genes; however, for comparisons between the control and high-osmotic stress samples, no gene had a *p*-value less than 0.05 after adjustment; thus, the *p*-value before adjustment was used for the selection. (2) The genes whose expression was higher than twofold under low-temperature and high-osmotic stress conditions compared to the control were selected as upregulated genes. (3) There was no gene whose expression was lower than twofold under low-temperature and high-osmotic stress conditions compared to the control; therefore, the gene whose expression was lower than twofold under high-osmotic stress conditions compared to that in the control was selected as the downregulated gene. Consequently*, cj1500*, *cj1254*, *cj1040*, and *cj0077* were selected as genes with higher expression levels in the VBNC state than in the controls. *cj1031* was selected as a downregulated gene whose expression level was more than 1.5-fold lower under low-temperature conditions compared to that in the control. The functions of each gene are listed in Table 1.

### Changes in gene expression during induction of the VBNC state

**Fig. 4:**
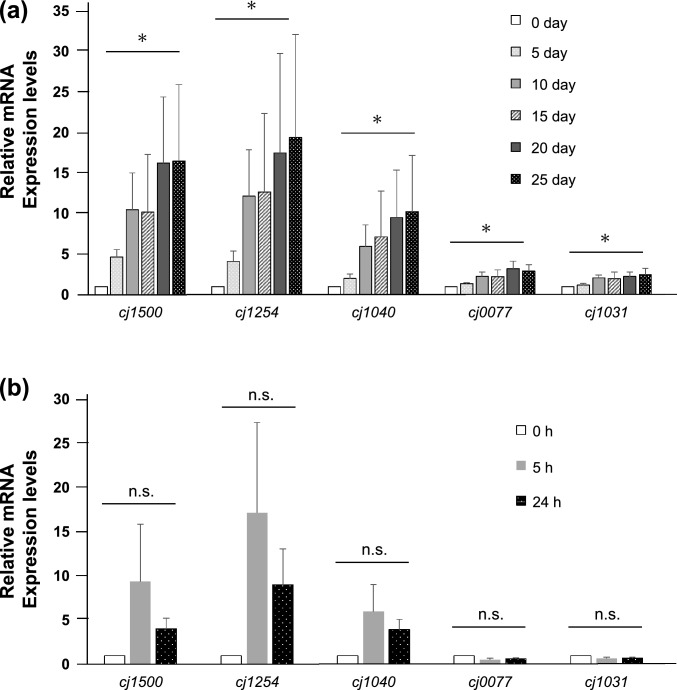
Changes in the expression of *cj1500*, *cj1254*, *cj1040*, *cj0077* and *cj1031* during induction of the VBNC state under low-temperature (**a**) and high-osmotic stress (**b**) conditions. Expression levels of each gene were expressed relatively as the ratio at 0 day (**a**) or 0 h (**b**). Data show the mean ± SE of three independent experiments. * = *p* < 0.05. n.s., not significant; qPCR, quantitative real-time polymerase chain reaction; SE, standard error; VBNC, viable but non-culturable

The expression of target genes under low-temperature and high-osmotic stress conditions was evaluated by qPCR using newly collected samples, including three low-temperature samples and three high-osmotic stress samples (Fig. 4). Under low-temperature conditions, the expression levels of *cj1500, cj1254, cj1040, cj0077,* and *cj1031* were significantly higher in the VBNC state (*p* < 0.05). Among these, *cj1254* exhibited the highest expression, followed by *cj1500*, *cj1040*, and *cj0077.* The expression level of *cj1031* under low-temperature condition, which was low in the microarray analysis, was higher in the qPCR analysis compared to the control samples (*p* < 0.05). No genes were significantly upregulated under high-osmotic stress. The expression levels of *cj1500*, *cj1254*, and *cj1040* tended to be higher, although the differences were not significant. The results of gene expression evaluation by microarray and qPCR analyses are summarised in Table 2.

**Table 2:** Changes in gene expression in the VBNC state

Gene	Microarray		qPCR	
	Low temperature	High-osmotic pressure	Low temperature	High-osmotic pressure
*cj1500*	+ +	+ +	+ +	+
*cj1254*	+ +	+ +	+ +	+
*cj1040*	+ +	+ +	+ +	+
*cj1031*	+ +	+ +	+ +	±
*cj0077*	–	–	+ +	±

+  + , significantly increased expression (*p* < 0.05); + , increased trend in expression (No significant difference); ± , No change in expression; –, decreased in expression (*p* < 0.05); qPCR, quantitative PCR; VBNC, viable but non-culturable

### Enzyme assays

The qPCR results showed that the expression of *cj1500*, *cj1245*, and *cj1040* was particularly high in the VBNC state. To investigate whether increased mRNA expression affects protein activity, the activity of FDH, which is associated with *cj1500*, was examined.

**Fig. 5:**
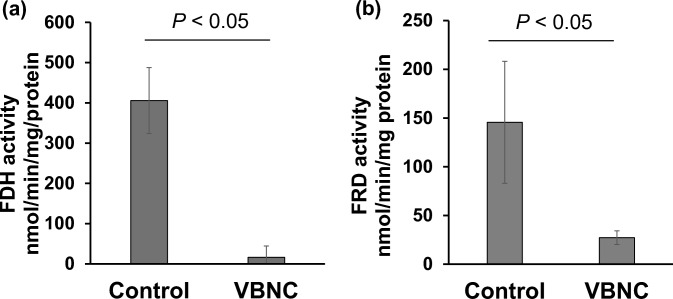
Formate dehydrogenase and fumarate reductase assay. Comparison of FDH (**a**) and FRD (**b**) activities between the control samples and the samples in the VBNC state induced by low-temperature conditions. Data show mean ± SD of three independent experiments. In the VBNC state, FDH activity significantly decreased. FDH, formate dehydrogenase; FRD, fumarate reductase; SD, standard deviation; VBNC, viable but non-culturable

The FDH activity was significantly reduced in the VBNC condition induced under low-temperature conditions compared to that in control (Fig. 5a). The enzyme activity of the samples VBNC state induced under high-osmotic stress conditions was also reduced, however, it was not shown because it is difficult to confirm whether the reduced activity was due to the many dead bacterial cells. To determine whether this decrease was specific to FDH, the FRD activity was also evaluated (Fig. 5b). FRD was selected because microarray results showed that the FRD-related genes *cj0408* and *cj0409* were upregulated as well as *cj1500* in the VBNC state. FRD activity was significantly reduced in the VBNC states.

## Discussion

In this study, to identify the *C. jejuni* genes involved in the induction of the VBNC state, the VBNC state was induced using low-temperature and high-osmotic stress conditions, and mRNA expression was quantified by microarray and qPCR analyses. Generally, data normalisation with respect to the expression of putative internal control genes is essential for qPCR analyses. Thus, the use of a suitable internal control gene that is stably expressed under the experimental conditions is important in such studies. Previously, *glyA*, *hipO*, and *rpoA* have been reported to be suitable internal controls for *C. jejuni* (LaGier et al. 2004; Ritz et al. 2009). Although *rpoA* has been used to quantify gene expression in *C. jejuni* under environmental stresses such as oxidative stress (Ritz et al. 2009), its mRNA levels were different between the control and VBNC samples (Table S3 and S4). Therefore, a new internal control gene was selected to develop a more accurate qPCR method. The most stable gene, *Pal*, was selected as an internal control by analysing qPCR data. While some studies have focused on narrowing down the internal control genes based on gene function, this study comprehensively validated these genes through microarray analysis. The new internal control gene may be useful not only for this study but also for other gene expression studies of *C. jejuni*.

Under high-osmotic stress, no significant changes in gene expression were observed in microarray or qPCR analysis. We hypothesized that it might be related to the low bacterial viability (Fig. 1). Although it was predicted that RNA degradation might be involved in the variety of the gene expression levels, bioanalyser results suggested that the effect of degradation was small (Fig. S1). It is possible that some conditions, rather than RNA degradation, affected RNA stability, and a method which can induce VBNC state with high viability should be used for exploring the mRNA involved in the induction of VBNC state. The expression levels of *cj1500*, *cj1254*, *cj1040*, *cj1031,* and *cj0077* were significantly higher in the VBNC state than those in culturable *C. jejuni*, indicating that *C. jejuni* may regulate its gene expression during the transition to the VBNC state. However, this study confirmed cell viability through the presence of cell membranes; thus, we tried CTC/DAPI staining to confirm the metabolic activity of VBNC cells at day 25, and almost all cells (90% <) indicated CTC positive (data not shown). Additionally, the microarray results were not consistent with the qPCR result; the expression of *cj1031* was significantly lower in the VBNC state than in culturable *C. jejuni* in the microarray results. In microarray analyses, 8–9 probes were designed for each gene; however, different results were sometimes observed with each probe for the same gene as described in a previous study (Chuaqui et al. 2002). Therefore, verification with qPCR is essential for confirming the microarray result.

The microarray and qPCR results are summarised in Table 2. Three genes with particularly large increases in transcription were *cj1500*, *cj1254*, and *cj1040*. Among these genes, we focused on *cj1500*, because Cj1500 compose FDH, which is related to respiratory metabolism, and some bacteria have been reported to resuscitate when respiratory substrates are added to the medium. For example, *Legionella pneumophila* can be resuscitated by the addition of sodium pyruvate, a respiratory substrate, to the medium (Ducret et al. 2014). In addition, *cj1500* might be important for the induction of the VBNC state because Kassem et al. 2012 showed that FDH was implicated in VBNC formation in *C. jejuni*, and Wulsten et al. 2020 showed that the addition of H_2_ at low oxygen levels substantially resuscitated *C. jejuni* from the VBNC state induced in raw milk.

Analysis of FDH activity using a benzyl viologen-coupled colorimetric assay showed decreased FDH activity in the VBNC state, although increased *cj1500* expression was observed. The results indicated that protein function does not necessarily correlate with gene transcription. Because we did not check the protein amount of Cj1500, it was not clarified whether the FDH composing protein amount is low or FDH activity is low. Western blotting or other experiments should be done to verify the actual protein amount of Cj1500. Therefore, our results suggested that stress exposure may shut down protein expression/activity and mRNA levels do not match. As shown in Table 3, *C. jejuni* has some genes annotated as FDH subunits (Shaw et al. 2012), and microarray results showed that the expression of *cj1514* in the VBNC state was lower than that in the culturable state. These suggested that bacteria upregulate the mRNA expression of some genes to re-grow as soon as the stress is removed; however, they suppress the metabolic activity by downregulating the mRNA expression of other genes. To investigate whether the reduction in FDH activity is a specific phenomenon, a similar experiment was conducted using another metabolic enzyme, FRD. FRD is a membrane-bound enzyme that catalyses the reduction of fumarate to succinate (Guccione et al. 2010). In the microarray results, the FRD-related genes *cj0408* and *cj0409* were upregulated as well as *cj1500* in the VBNC state. However, FRD activity was downregulated in the VBNC state in *C. jejuni*. Downregulation of mRNA expression of one or some genes related to FRD activity might supress the protein activity of FRD in the same way as FDH.

**Table 3:** The genes related FDH activity

Gene ID	Function	Microarray fold change (low temperature)	Microarray fold change (high-osmotic pressure)
*cj1500*	fdhT (FDH subunit)	+	+
*cj1501*	fdhU (FDH subunit)	±	±
*cj1507*	regulator	±	±
*cj1508*	fdhD (FDH subunit)	±	±
*cj1509*	fdhC (FDH subunit)	±	±
*cj1510*	fdhB (FDH subunit)	±	±
*cj1511*	fdhA (FDH subunit)	+	±
*cj1513*	cofactor	±	±
*cj1514*	fdhM (FDH subunit)	–	–

+ , increased expression (*p* < 0.05); ± , No change in expression; –, decreased in expression (*p* < 0.05)

This study identified three mRNAs whose expression increased during the induction of the VBNC state in *C. jejuni*. Thus, the findings indicated that *C. jejuni* actively changed its mRNA expression during induction of the VBNC state, where they inhibit the metabolic activity by suppressing some gene expression. This study may contribute to further research aimed at clarifying the molecular mechanisms involved in the induction of the VBNC state in *C. jejuni.*

### Supplementary Information

Below is the link to the electronic supplementary material.

Supplementary file1 (XLSX 11 KB)

Supplementary file2 (XLSX 10 KB)

Supplementary file3 (XLSX 127 KB)

Supplementary file4 (XLSX 18 KB)

Supplementary file5 (PPTX 3652 KB)

## Data Availability

The datasets generated during and/or analysed during the current study are available from the corresponding author upon reasonable request.
