# Supplementary material for: Exploration of genes associated with induction of the viable but non-culturable state of Campylobacter jejuni
Source: Arch Microbiol. 2024 May 15;206(6):260. doi: 10.1007/s00203-024-03980-y (PMC11093796; doi:10.1007/s00203-024-03980-y)
Supplement: Supplementary file 5 — Supplementary file5 (PPTX 3652 KB) [file 203_2024_3980_MOESM5_ESM.pptx]

## Slide 1
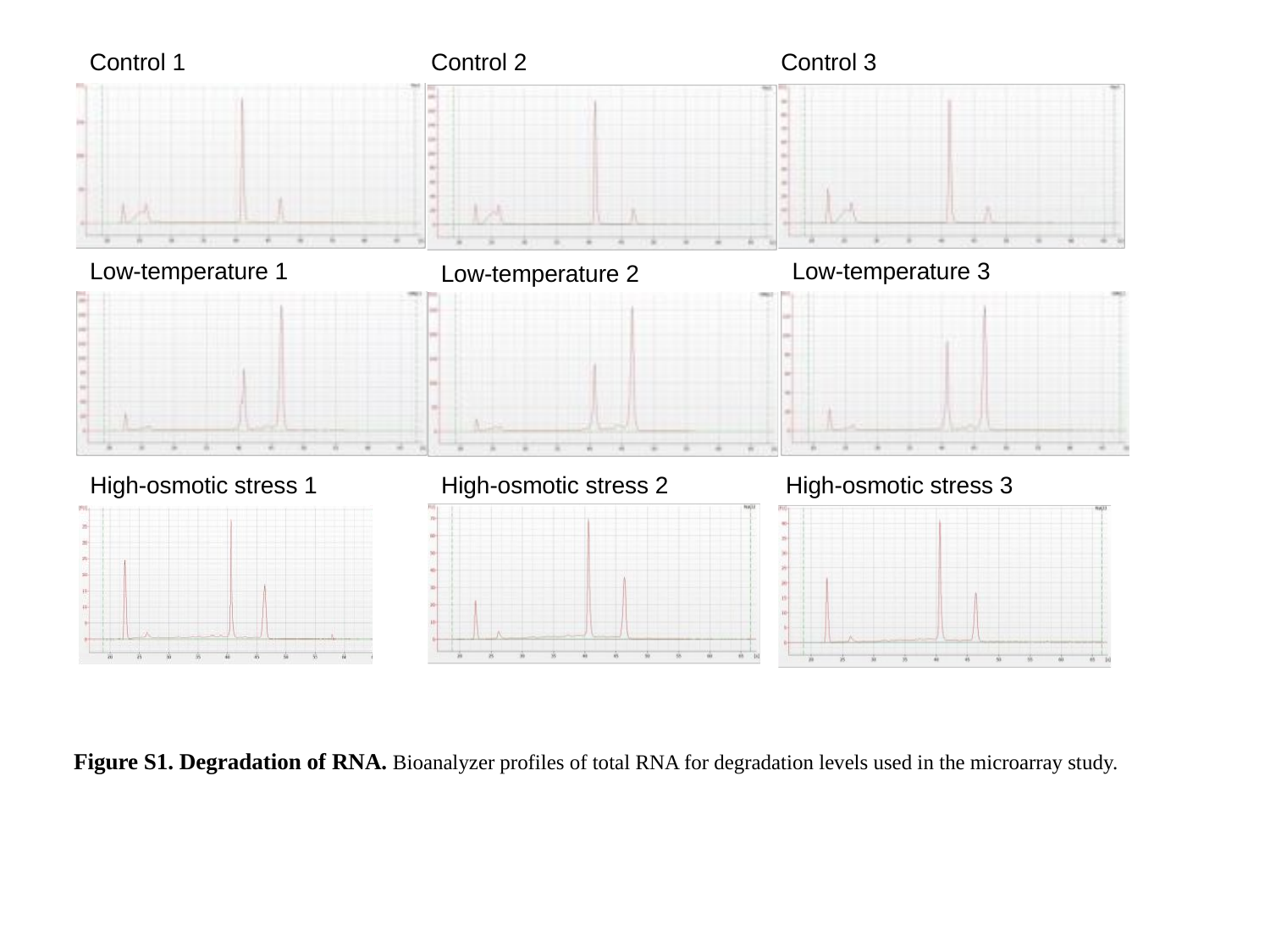

Control 1
Control 2
Control 3
Low-temperature 1
Low-temperature 3
Low-temperature 2
High-osmotic stress 1
High-osmotic stress 2
High-osmotic stress 3
Figure S1. Degradation of RNA. Bioanalyzer profiles of total RNA for degradation levels used in the microarray study.
